# 无鞘流毛细管电泳-电喷雾串联质谱用于面粉中荧光增白剂的高灵敏检测

**DOI:** 10.3724/SP.J.1123.2023.11023

**Published:** 2024-06-08

**Authors:** Anping WANG, Chushi CHEN, Jinlan YANG, Li YANG

**Affiliations:** 东北师范大学, 吉林 长春 130024; Northeast Normal University, Changchun 130024, China

**Keywords:** 毛细管电泳, 无鞘流电喷雾电离串联质谱, 荧光增白剂, 面粉, capillary electrophoresis (CE), sheathless electrospray ionization-tandem mass spectrometry (sheathless ESI-MS/MS), fluorescent whitening agents (FWAs), flour

## Abstract

食品中残留的过量荧光增白剂(FWAs)对人体健康存在潜在威胁,因此,开发准确、高灵敏的FWAs检测方法在食品安全监测方面具有重要意义。本工作提出了利用无鞘流接口的毛细管电泳-电喷雾串联质谱(CE-ESI-MS/MS)的方法,以实现面粉样品中6种FWAs的高灵敏检测。实验采用超声辅助液相萃取法进行样品前处理,以减小复杂样品中基质效应的干扰。以氯仿-甲醇(3∶2, v/v)溶液作为萃取剂,在30 ℃下对样品中的FWAs进行萃取。萃取完成后,经离心、氮气吹干后用氯仿-甲醇(1∶4, v/v)复溶后检测。无鞘流CE-ESI-MS/MS方法采用正离子(ESI^+^)和多反应监测(MRM)模式,利用二级质谱对6种目标物同时进行定性定量分析,从而提高方法的检测通量和灵敏度。结果显示,本方法具有较宽的线性范围、良好的线性关系和较低的方法检出限(0.04~0.67 ng/g),在实际样品中3个水平下的加标回收率良好(86.2%~103.7%),日间和日内重复性(RSD)分别不大于11.5%和10.2%。上述研究表明,本方法适用于复杂基质中多种FWAs的准确、高灵敏检测,在面粉样品的质量评估和FWAs的污染监控方面具有潜在的应用价值。

## Abstract (en)

Fluorescent whitening agents (FWAs) are dyes that emit visible blue or blue-purple fluorescence upon ultraviolet-light absorption. Taking advantage of light complementarity, FWAs can compensate for the yellow color of many substances to achieve a whitening effect; thus, they are used extensively in various applications. FWAs are generally stable, but their presence in the environment can lead to pollution and accumulation in the body through the food chain. Recent studies have revealed that some types of FWAs, such as coumarin-based FWAs, may exhibit photo-induced mutagenic effects that can trigger allergic reactions in humans and even pose carcinogenic risks. Hence, the development of an accurate and highly sensitive method for detecting FWAs in food-related samples is a crucial endeavor. Owing to the high polarity and structural similarity of FWAs, the accurate determination of these substances in complex food samples requires an analytical method that offers both efficient separation and sensitive detection. Capillary electrophoresis (CE) exhibits essential features such as high separation efficiency, short analysis times, very small sample injection requirements, minimal use of organic solvents, and simple operation. Thus, it is often used as an effective alternative to liquid chromatographic techniques. Over the past few decades, electrospray ionization mass spectrometry (ESI-MS) has been utilized as a highly sensitive and accurate detection method in numerous chemical analytical fields because it enables the analysis of molecular structures. By combining the high separation efficiency of CE with the high sensitivity of ESI-MS, a powerful tool for identifying and quantifying trace amounts of FWAs in food samples may be obtained. In this study, we present a method based on sheathless CE coupled with electrospray ionization tandem mass spectrometry (ESI-MS/MS) for the simultaneous detection of six trace FWAs in flour. In the proposed method, the CE separation device is directly coupled to the mass spectrometer through a sheathless interface without the need for a sheath liquid for electric contact, thereby avoiding the dilution of the analytes and improving detection sensitivity. Various conditions that could affect extraction recovery, separation efficiency, and detection sensitivity were evaluated and optimized. The FWAs were effectively extracted from the sample matrix with reduced matrix effects by ultrasonic-assisted extraction at a temperature of 30 ℃ for 20 min using CHCl_3_-MeOH (3∶2, v/v) as the extraction solvent. The extract was centrifuged, dried under N_2_, and reconstituted in CHCl_3_-MeOH (1∶4, v/v) for subsequent analysis. During the detection process, the CE device was coupled to the ESI-MS/MS instrument via a highly sensitive porous spray needle, which served as the sheathless electrospray interface. The target FWAs were scanned in positive-ion mode (ESI^+^) to ensure the stability and intensity of the obtained signals. Additionally, multiple-reaction monitoring (MRM) mode and MS/MS analysis were used to simultaneously quantify the six targets with high selectivity. The developed sheathless CE-ESI-MS/MS method detected the FWAs with high sensitivity over wide linear ranges with low method limits of detection (0.04-0.67 ng/g). The recoveries of the six target FWAs at three spiked levels were between 77.5% and 97.2%, with good interday (RSD≤11.5%) and intraday (RSD≤10.2%) precision. Analyses of the six target FWAs in eight commercial flour samples were performed using this method, and four positive samples were identified. These results demonstrate that the proposed CE-ESI-MS/MS method is a promising strategy for the determination of trace FWAs in complex food sample matrices with efficient separation and high sensitivity.

荧光增白剂(FWAs)是一类含有芳香环和取代基的复杂有机物,其结构中形成的连续共轭体系在吸收紫外光后可激发出可见的蓝色或蓝紫色荧光^[1]^。利用光的互补性,FWAs能够实现去黄增白的效果,而不会对基质造成损害,因此被广泛应用于纸制品、洗涤剂、包装材料等产品中^[2⇓⇓-5]^。然而多数情况下,FWAs的结构稳定,不易降解或代谢。因此,环境中存在的FWAs会导致持久污染,甚至通过食物链在人体中积累^[6⇓-8]^。研究表明,某些类型的FWAs具有光诱导诱变作用,可引起人类的过敏反应,甚至具有潜在的致癌性^[9,10]^。鉴于这些潜在风险,世界各地的国家食品安全部门严格禁止在食品或食品接触材料中添加FWAs^[11⇓-13]^。因此,对食品样品中FWAs实现同时准确的灵敏检测至关重要。然而,由于不同种类的FWAs往往具有较高的极性和相似的结构,复杂食品样品中FWAs的检测需要兼具高效的分离能力和较高的灵敏度^[14,15]^。近年来,人们不断开发出针对复杂样品中FWAs的检测方法^[16,17]^。毛细管电泳(CE)是一种高效分离技术,具有分离效率高、分析时间短、进样量极低、有机溶剂消耗量少以及操作简单等特点^[18⇓⇓-21]^,被认为是液相色谱技术的有效补充方法。一些研究利用CE结合紫外(UV)或激光诱导荧光(LIF)检测,对食品和食品包装材料中的几种FWAs进行分析^[22⇓-24]^。虽然基于CE-UV和CE-LIF的检测方法可以高效分离FWAs,但检测灵敏度相对较低,且对成分结构的鉴别能力不足。在过去的几十年里,电喷雾电离质谱(ESI-MS)凭借灵敏度高、分析质量范围广以及强大的定性能力,被广泛应用于分析化学的许多领域^[25⇓⇓-28]^。CE-ESI-MS结合了CE的高分离效率和ESI-MS的高灵敏度和分子鉴定能力,尤其适用于食品样品中微量FWAs的定性和定量分析。然而,据我们所知,目前尚无CE-ESI-MS技术在FWAs检测方面的相关报道。

本工作利用毛细管电泳-无鞘流电喷雾电离串联质谱(无鞘流CE-ESI-MS/MS)联用技术,提出一种用于面粉样品中FWAs的高灵敏检测方法。在无鞘流CE-ESI-MS/MS技术中,分析物经CE分离后通过无鞘流界面直接实现质谱的电喷雾离子化和检测。无鞘流型接口不需要鞘流进行电接触,有效避免了分析物的稀释,显著提高了检测灵敏度。研究结果表明,无鞘流CE-ESI-MS/MS技术不仅具备高效的分离能力,还具有高灵敏度,在复杂食品样品基质中微量FWAs的检测方面展现出潜在的应用价值。本文系统考察和优化了可能影响萃取回收率、分离效率和检测灵敏度的相关影响因素,并在最优条件下对该分析方法的分析性能做出了评估,成功实现了8种商业面粉样品中6种FWAs的同时定性定量分析。

## 1 实验部分

### 1.1 仪器、试剂与材料

毛细管电泳仪(CESI 8000 plus)与质谱仪(Triple TOF 4600)(AB Sciex, USA)。

6种荧光增白剂标准品7-二乙氨基-4-甲基香豆素(FWA52)、1,2-双(5-甲基-2-苯并恶唑基)乙烯(FWA135)、2,5-双(5-叔丁基-2-苯并恶唑基)噻吩(FWA184)、2,5-双(苯并恶唑-2-基)噻吩(FWA185)、1,4-双(2-苯并恶唑基)萘(FWA367)、4,4-双(2-苯并恶唑基)二苯乙烯(FWA393)(纯度均>98%)以及三氯甲烷(CH_3_Cl)均购自鼎国生物技术有限公司(北京)。色谱纯氨水、乙腈和醋酸铵(NH_4_Ac)购自阿拉丁生化科技股份有限公司(上海),甲醇(色谱纯)购自赛默飞世尔有限公司(上海)。所有实验用水均为使用Milli-Q超纯水系统净化制得的超纯水。CE系统中使用的两根熔融硅毛细管均购自英诺生物技术有限公司(深圳)。

实际样品检测中使用的8种面粉样品(面包粉1种、小麦粉1种、高筋面粉2种、中筋面粉1种、雪花粉3种)均购自当地超市,密闭保存在离心管中置于阴凉避光处备用。

### 1.2 溶液配制

6种荧光增白剂的储备溶液(1 g/L)由氯仿-甲醇(1∶4, v/v)溶解配制,并置于棕色玻璃瓶中4 ℃密封保存,使用前用甲醇稀释到所需浓度。CE缓冲体系:以甲醇-乙腈 (4∶1, v/v)溶解的20 mmol/L醋酸铵溶液,并用氨水调节至pH=10。试验试剂实验当天配制,使用前超声10 min并用孔径为0.22 μm的聚醚砜(PES)膜过滤。

### 1.3 无鞘流CE-ESI-MS/MS检测条件

毛细管电泳仪与质谱仪通过无鞘流型电喷雾接口实现联用,进行所有后续的分析实验。

总长为90 cm的熔融硅毛细管(30 μm i. d., 150 μm o. d.)用于CE分析,其有效分离长度为85 cm。毛细管出口端设计为多孔尖端,作为无鞘流CE-ESI-MS/MS的无鞘流接口界面。多孔尖端的制备及其与ESI-MS的耦合过程与我们之前的研究^[28]^类似,具体步骤如下:将毛细管的一端用砂纸打磨光滑齐平,之后在距离其末端5 cm处,通过火焰剥离去除聚酰亚胺涂层,用乙醇将其外壁擦拭干净。将处理好的毛细管插入含有6 mL新配制的48%(质量分数)HF溶液和100 μL辛醇的10 mL离心管中,并持续向毛细管内通入N_2_。离心管中上层的辛醇可以避免HF腐蚀未去除涂层部分毛细管外壁,N_2_的持续通入可以防止HF通过内部虹吸作用刻蚀毛细管内壁,避免毛细管在腐蚀过程中堵塞。HF刻蚀过程在通风橱中完成,并保持温度为恒温25 ℃。当HF刻蚀时间持续约20 min后,将毛细管尖端部分依次浸入2.5 mol/L NaOH中2 h、超纯水中3 h,以去除刻蚀过程中毛细管壁残留的多余酸和碱。

CE系统由分离毛细管和导电液毛细管组成。以经末端多孔刻蚀的90 cm毛细管作为分离毛细管,导电液毛细管选用未处理的80 cm熔融石英毛细管(50 μm i. d., 365 μm o. d.),二者均安装在Opti MS保护卡盒中。CE分析在25 ℃恒温条件下进行,分离电压为+25 kV,辅助压力为34.5 kPa,进样条件为10 s×34.5 kPa,在分离毛细管和导电液毛细管中均充入CE缓冲体系。采用正离子模式(ESI^+^)及多反应监测(MRM)模式进行质谱检测,具体参数如下:离子喷雾电压(ISVF)1600 V,界面加热温度50 ℃, gas 1/gas 2/气帘气分别设置为0/0/0.06 MPa。

在无鞘流CE-ESI-MS/MS分析运行之前,在0.69 MPa的压力下依次用甲醇(10 min)、水(5 min)、0.1 mol/L NaOH溶液(2 min)和水(5 min)冲洗和平衡分离毛细管,然后用缓冲体系冲洗10 min。在两次运行之间,分离毛细管在0.69 MPa压力下分别用0.1 mol/L NaOH溶液、水和缓冲体系冲洗4 min,导电液毛细管在0.5 MPa压力下用缓冲体系冲洗3 min。每天实验结束后,分离毛细管和导电液毛细管分别在0.69 MPa和0.5 MPa压力下用水和甲醇分别冲洗5 min。

### 1.4 样品预处理

本文采用超声辅助萃取法对面粉样品进行处理。准确称取固体面粉样品各0.5 g于10 mL玻璃离心管中,加入5 mL 氯仿-甲醇 (3∶2, v/v)涡旋混匀1 min,将盛有混合溶液的玻璃离心管放入超声波清洗器中,在30 ℃条件下超声萃取20 min。萃取结束后,上述混合物在5000 r/min下离心10 min,收集的上清液经有机滤膜过滤、N_2_吹干后再用氯仿-甲醇(1∶4, v/v)溶解,置于4 ℃条件下避光保存以备后续无鞘流CE-ESI-MS/MS检测。

### 2结果与讨论

#### 2.1 FWAs萃取条件优化

高效提取样品中的目标物对于降低基质效应以及提高方法检测灵敏度至关重要。依据“相似相溶”的原则,本工作采用超声辅助萃取法对待测物质进行萃取,通过超声波增大物质分子的运动频率和速度,提高溶剂穿透力,加速目标成分进入溶剂,从而加速萃取过程。为获得最佳的萃取效果,我们对萃取剂的种类、用量、萃取时间和萃取温度进行了优化。

6种目标FWAs中,FWA393和FWA367仅溶于氯仿,其余4种FWAs易溶于甲醇和乙腈。因此,本研究采用氯仿-甲醇或氯仿-乙腈的混合溶液作为萃取溶剂。为了最大限度地提高回收率并尽量减少有机溶剂的用量,我们考察了氯仿-乙腈(2∶3, v/v,下同)、氯仿-甲醇(2∶3)、氯仿-乙腈(3∶2)、氯仿-甲醇(3∶2)、氯仿-乙腈(4∶1)、氯仿-甲醇(4∶1)6种溶剂对加标样品中待测物质的萃取效果。如图1a所示,当选择氯仿与乙腈混合溶液作为萃取溶剂时,FWA393的回收率始终低于65%,这可能是由于FWA393在乙腈中溶解度较差导致。使用氯仿与甲醇混合溶液作为萃取溶剂时FWA393的回收率明显提高,且在氯仿-甲醇(3∶2)时各物质萃取效果最佳(回收率83%~97%),故选择氯仿-甲醇(3∶2)作为最优萃取溶剂。萃取溶剂体积的优化结果显示(图1b),在添加20 ng/g FWAs混合标准品的0.5 g面粉样品中,5 mL萃取溶剂体积获得了最佳的回收率。图1c、1d显示了萃取时间和温度对每个目标物回收率的影响。图1c结果显示,各目标物的回收率在20 min内逐渐增加,继续延长时间对物质萃取没有明显促进效果。图1d的结果表明,当萃取温度≥30 ℃时,各目标物的回收率达到最优值。综上,得到优化后的萃取条件(见1.4节)。

**图 1:**
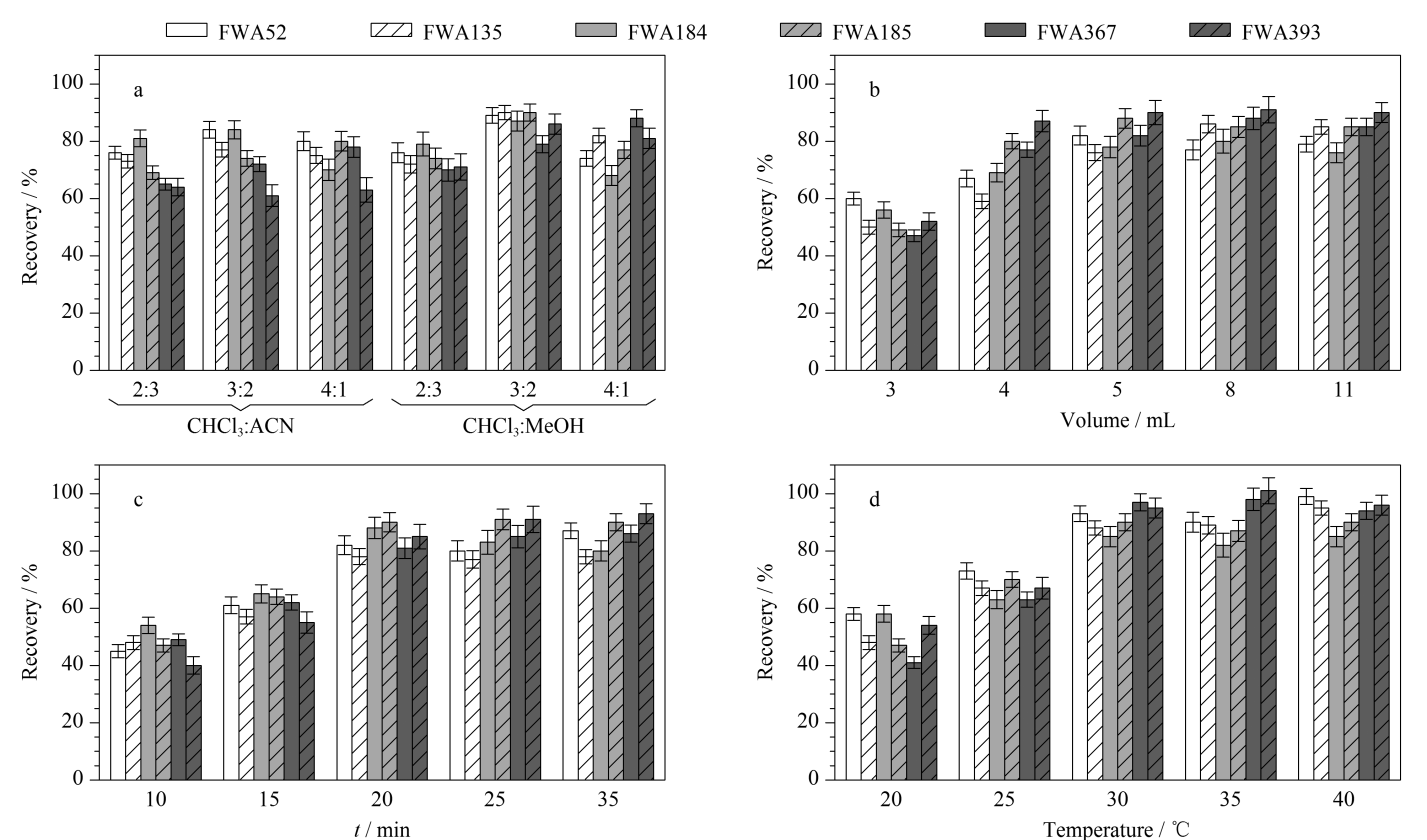
萃取条件对FWAs回收率的影响(*n*=3)

#### 2.2 CE分离与无鞘流ESI-MS/MS条件优化

为了实现样品中FWAs的灵敏检测,首先优化了质谱检测条件。将FWAs标准溶液通过Nano ESI接口注入质谱仪,并采用全扫描模式分析,扫描范围为*m/z* 50~500。对比了质谱正、负离子模式下的信号强度,结果显示正离子模式下信号强度远高于负离子模式,故选择正离子模式进行后续的检测。在子离子扫描模式下,对每种目标物的去簇电压(DP)和碰撞能量(ColE)分别进行了优化,以增强各母离子的碎片峰在二级质谱模式下的信号强度。每种物质产生的两种碎片离子峰分别用于定性和定量。表1详细列出了6种目标化合物的结构信息和优化的质谱参数。在最优条件下,各物质定量和定性特征峰如图2所示。

**表 1:** 6种目标FWAs的结构信息和ESI-MS/MS参数

FWA	Molecular formula	Chemical structure	[M+H]^+^	DP/V	ColE/eV
FWA52	C_14_H_17_NO_2_	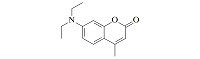	232.1650	30	40
FWA135	C_18_H_14_N_2_O_2_	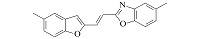	291.1510	30	40
FWA184	C_26_H_26_N_2_O_2_S	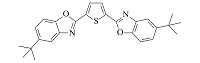	431.2328	40	40
FWA185	C_18_H_10_N_2_O_2_S	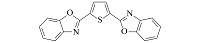	319.0953	40	45
FWA367	C_24_H_14_N_2_O_2_	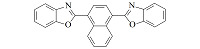	363.1595	50	40
FWA393	C_28_H_18_N_2_O_2_	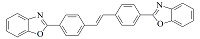	415.2624	50	45

DP: declustering potential; ColE: collision energy.

**图 2:**
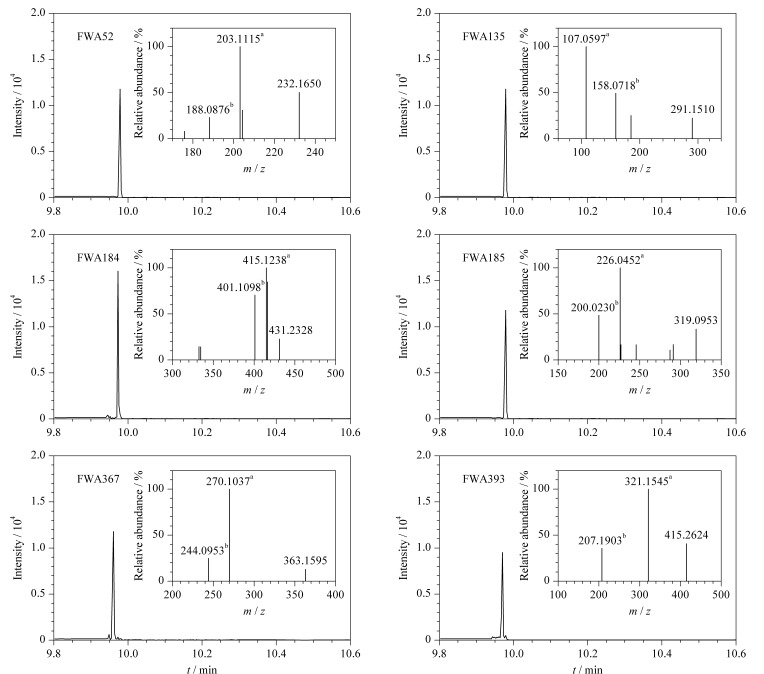
无鞘流CE-ESI-MS/MS检测6种FWAs的提取离子流图

为了提高方法的分析性能,考察和优化了影响CE分离的一些重要因素,包括缓冲体系的组成、pH和分离辅助压力等。由于MS分析需要低电导率、易挥发的背景体系,故本实验选择了有利于ESI离子化的常用缓冲体系醋酸铵作为初始的缓冲体系,并考察了其酸碱性对ESI离子化效率的影响。首先对比了使用酸性缓冲体系(20 mmol/L醋酸铵以甲醇(添加20% (体积分数)HAc)溶解(pH=4.3))和碱性缓冲体系(20 mmol/L醋酸铵以甲醇溶解后用NH_3_·H_2_O调至pH=10)进行测试时得到的总离子流图的信号强度。结果显示,使用酸性缓冲体系对信号产生了明显的抑制,而采用相同浓度碱性缓冲体系时信号强度高、峰形对称且分析速度更快。这是由于在两种缓冲体系条件下,6种目标物均带负电,而高pH的缓冲体系电渗流更大,使得碱性缓冲体系下峰的迁移时间更短。因此,我们选择碱性缓冲体系进行后续实验。

系统考察了碱性缓冲体系的pH值、乙腈添加量及分离辅助压力对各物质信号强度和峰形的影响。如图3a所示,随着缓冲体系pH的升高,各物质的色谱峰变窄,但当pH=11时各物质峰形出现分叉,这可能是由于ESI在碱性较高的条件下喷雾不稳定造成的,而pH=10条件下各物质峰形最佳。为进一步改善喷雾效果并获得更高的检测信号,我们向缓冲体系中添加了不同体积分数的乙腈,如图3b所示,当乙腈体积分数为20%时,5-FWA367和6-FWA393两种物质的信号峰强度有明显改善,继续增加乙腈的体积分数对信号无明显影响,故选择添加20%作为乙腈的最佳体积分数。此外我们对比了20.7、34.5、61.0 kPa分离辅助压力下的对检测信号,结果表明分离辅助压力为34.5 kPa时,各物质的峰形和信号强度达到最佳。综上,我们选择以甲醇-乙腈 (4∶1, v/v)溶解的20 mmol/L醋酸铵溶液,并用氨水调节至pH=10作为最优缓冲体系。pH=10,添加20%乙腈的醋酸铵缓冲体系,并在34.5 kPa分离辅助压力下进行检测。

**图 3:**
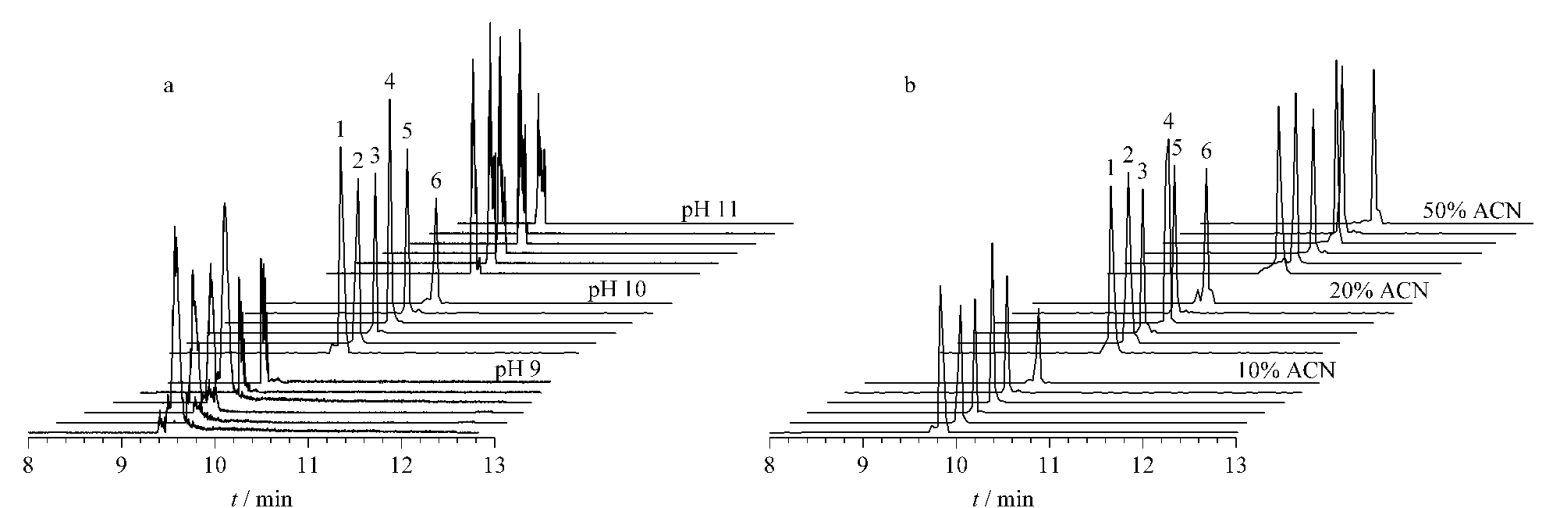
不同CE条件下6种荧光增白剂的提取离子流图

#### 2.3 分析性能评估

为评估本方法对面粉样品中6种荧光增白剂的分析性能,在优化的最佳实验条件下对6种荧光增白剂进行了分析。结果表明,本方法对各目标物均具有较宽的线性范围和良好的线性关系(*R*^2^≥0.989)(见表2)。通过对未经超声辅助萃取预处理的加标样品直接进行无鞘流CE-ESI-MS/MS分析,在信噪比*S/N*=3和*S/N*=10条件下分别对每种FWA的仪器检出限(iLOD)和定量限(iLOQ)进行了评估。如表2中结果显示,各目标物的iLOD与iLOQ分别为0.35~2.20 ng/g与1.00~6.50 ng/g。方法的检出限和定量限(mLOD, *S/N*=3和mLOQ, *S/N*=10)通过对加标样品进行超声提取后的无鞘流CE-ESI-MS/MS分析结果评估,分别为0.04~0.67 ng/g与0.20~2.20 ng/g,表明该方法具有较高的灵敏度,可以满足实际食品样品中FWAs的检测需求。日内和日间重复性分别通过计算一天内连续5次试验和连续5天试验结果的RSD值进行评估,结果显示峰迁移时间与峰面积的RSD值分别不大于9.1%和10.7%,表明方法具有良好的重复性。

**表 2:** 各物质的线性范围、回归方程、相关系数、mLODs、mLOQs、iLODs、iLOQs、ME及重复性

Compound	Linear range/ (ng/g)	Regression equation	R^2^	mLOD/ (ng/g)	mLOQ/ (ng/g)	iLOD/ (ng/g)	iLOQ/ (ng/g)	ME	Repeatabilities (RSDs/%)	
									T	A
FWA52	0.2-25	y=23.37x+93.29	0.989	0.07	0.20	0.55	1.75	1.04	7.3	9.7
FWA135	0.5-25	y=20.26x+22.35	0.990	0.16	0.50	1.20	3.50	0.80	8.1	8.7
FWA184	0.3-25	y=154.49x+38.71	0.993	0.04	0.28	0.35	1.00	0.90	6.9	9.0
FWA185	0.5-50	y=23.38x+51.05	0.994	0.17	0.50	1.20	3.45	1.15	7.4	10.3
FWA367	0.5-50	y=10.63x+2.85	0.991	0.17	0.50	1.00	3.20	0.87	9.1	10.2
FWA393	2.2-100	y=59.83x+66.82	0.996	0.67	2.20	2.20	6.50	1.02	6.9	10.7

mLOD: limit of detection of methodology (*S/N*=3); mLOQ: limit of quantification of methodology (*S/N*=10); iLOD: instrumental limit of detection (*S/N*=3); iLOQ: instrumental limit of quantification (*S/N*=10); RSDs: relative standard deviations (*n*=5); T: migration time; A: peak area.

由于面粉样品中基质成分复杂,为了减少基质对测定结果的影响,我们对基质效应(ME)进行了评估。通过在空白面包粉基质和溶剂氯仿-甲醇(1∶4, v/v)中分别添加不同浓度的FWAs混合标准品,并利用无鞘流CE-ESI-MS/MS检测,得出了基质背景下和溶剂中各物质的标准曲线。通过计算每种目标物质在空白基质(*k*_基质_)和溶剂(*k*_标准_)中的两条标准曲线的斜率之比进行ME的评估。6种荧光增白剂的ME范围为0.80~1.15(见表2),表明样品经超声辅助萃取处理后,其基质效应可以忽略。

回收率试验通过向空白面粉样品中加入荧光增白剂标准品作为分析样品来进行。为验证本方法在面粉实际样品检测中的可行性,我们在无FWAs的空白面粉样品中加入不同含量的混合标准品(1倍、2倍和10倍mLOQ)。如表3所示,6种FWAs的加标回收率为86.2%~103.7%,日内重复性(*n*=5)和日间重复性(*n*=5)分别不大于10.2%和11.5%。实际样品中较高的回收率和良好的重复性表明本方法适用于面粉样品中FWAs的同时准确检测。

**表 3:** 3种不同加标水平下方法的平均回收率和重复性

Compound	Added/(ng/g)				Recoveries (RSDs)/%(n=5)				Repeatabilities (RSDs)/%(n=5)	
	1×mLOQ	2×mLOQ	10×mLOQ		1×mLOQ	2×mLOQ	10×mLOQ		Intraday	Interday
FWA52	2.0	4.0	20.0		102.2 (7.4)	92.4 (9.2)	89.2 (8.7)		8.9	9.1
FWA135	4.5	9.0	45.0		90.4 (7.9)	89.5 (9.2)	101.4 (9.6)		10.2	11.5
FWA184	1.5	3.0	15.0		100.2 (6.9)	86.2 (7.2)	91.2 (9.0)		7.9	9.4
FWA185	5.0	10.0	50.0		95.3 (7.4)	101.9 (9.5)	92.6 (6.3)		8.6	10.2
FWA367	5.0	10.0	50.0		87.4 (6.5)	88.2 (8.7)	92.7 (7.8)		9.6	11.3
FWA393	15.0	30.0	150.0		103.7 (9.8)	88.6 (9.4)	87.5 (10.2)		7.8	10.7

采用所建立方法对市售的8种不同品牌的面粉中6种荧光增白剂进行了检测分析,在4种面粉样品中检出不同种类的荧光增白剂,其中FWA184在3个样品中均被检出,含量分别为20.35、 40.40和67.20 ng/g; FWA185和FWA393分别仅在一种样品中被检出,含量分别为60.50 ng/g和15.70 ng/g;其余3种FWAs均未在样品中检出。由于FWAs在生产过程中禁止作为食品添加剂使用,市售面粉样品中FWAs的阳性检测结果可能源于食品包装的迁移或非法添加。图4给出了4个阳性样品的提取离子流图。

**图 4:**
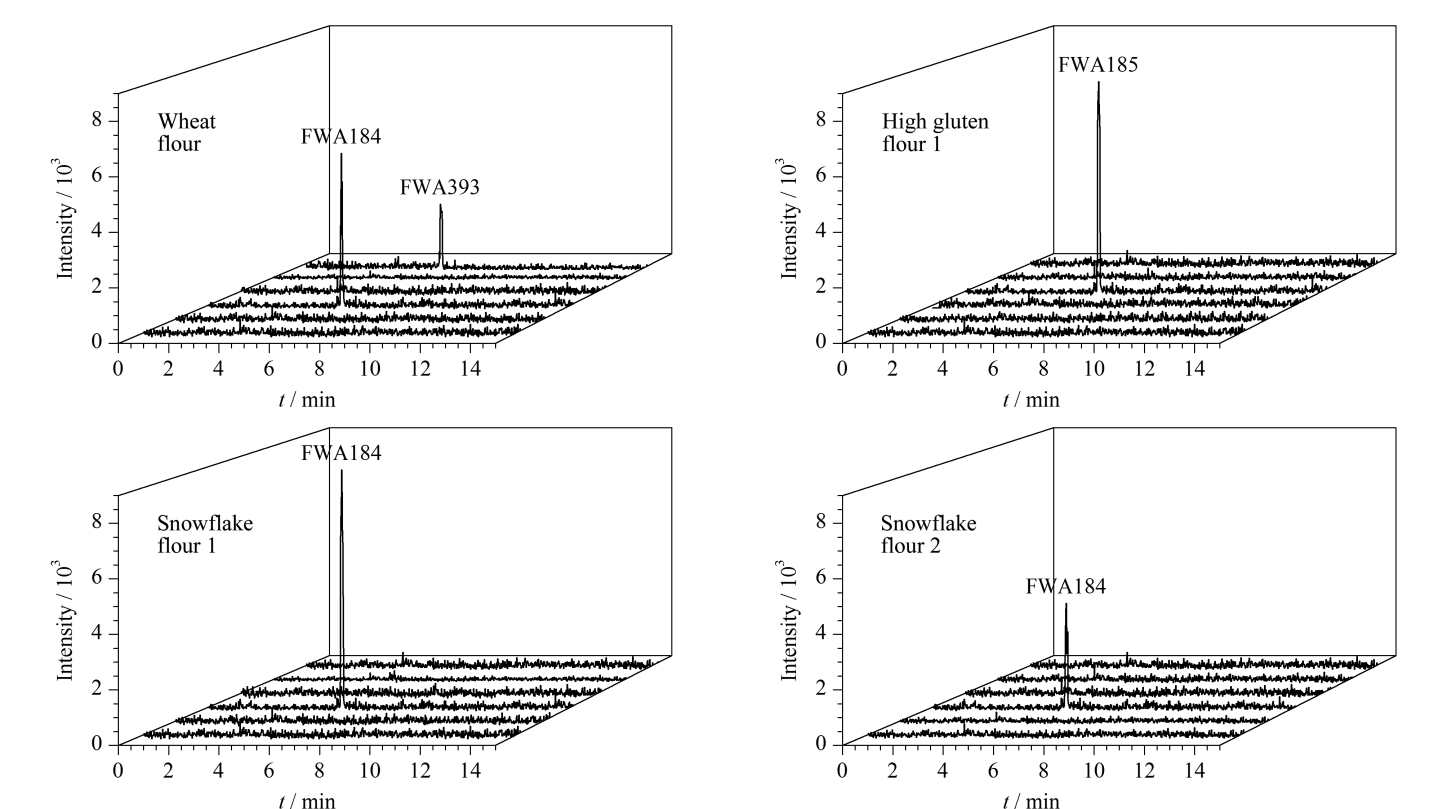
4种阳性面粉样品经无鞘流CE-ESI-MS/MS检测得到的提取离子流图

表4中,我们将无鞘流CE-ESI-MS/MS技术与其他技术对面粉中FWAs的检测性能进行了对比。

**表 4:** 本文方法与其他方法对面粉中FWAs的检测性能的比较

Injection volume/ μL	Analysis method	Pre- treat- ment	Target	LOD/ (μg/L)	Linear range/ (μg/L)	Ref.
5.0	UPLC-	UAE	FWA52	0.2^a^	0.2-20.0	[29]
	MS/MS		FWA135	0.2^a^	0.2-20.0	
			FWA185	0.5^a^	0.5-50.0	
			FWA393	2.0^a^	2.0-200	
			FWA367	0.5^a^	0.5-50.0	
			FWA368	2.0^a^	2.0-200	
			FWA184	0.2^a^	0.2-20.0	
10	HPLC-	LPE	FWA135	1.10	1.0-64.0	[14]
	MS/MS		FWA140	0.50	2.0-64.0	
			FWA162	1.75	0.2-64.0	
			FWA184	1.35	0.1-32.0	
			FWA185	0.70	0.2-64.0	
			FWA367	1.05	1.0-64.0	
			FWA393	2.65	1.0-64.0	
10	HPTLC-	UAE	FWA184	18	100-2000	[8]
	MS		FWA367	21	100-2000	
Solid	MM-IR	-	FWA393	10	-	[16]
0.001	sheathless	UAE	FWA52	0.07	0.2-25	this
	CE-ESI-		FWA135	0.16	0.5-25	work
	MS/MS		FWA184	0.04	0.3-25	
			FWA185	0.17	0.5-50	
			FWA367	0.17	0.5-50	
			FWA393	0.67	2.2-100	

a: LOQ; MM-IR: multi-molecular infrared spectroscopy; UAE: ultrasonic-assisted extraction; LPE: liquid phase extraction.

可以看出,得益于CE的高效分离和MS的高灵敏检测,采用本方法检测面粉样品中的FWAs具有较高的灵敏度和较宽的线性范围。此外,由于毛细管小体积的优势,单次检测所需的进样量低至纳升级,检测中消耗的试剂量也相对较少,符合绿色化学的理念。

## 3 结论

本工作建立了一种利用无鞘流CE-ESI-MS/MS实现食品样品中FWAs同时高灵敏检测的方法,系统考察了可能影响CE分离、质谱检测和超声波辅助萃取的相关实验条件,在最优条件下评估了该方法对6种FWAs的检测性能。结果显示,该方法能够在较宽的线性范围内对FWAs进行准确、灵敏的分析,具有较低的检出限和较高的重复性。该方法成功应用于商业面粉样品中6种FWAs的同时定性定量分析。上述结果表明,CE与无鞘流ESI-MS/MS的结合能够实现食品样品中微量FWAs的准确定量分析,在食品安全领域具有重要的应用价值。
